# Advanced oxidation of the commercial nonionic surfactant octylphenol polyethoxylate Triton™ X-45 by the persulfate/UV-C process: effect of operating parameters and kinetic evaluation

**DOI:** 10.3389/fchem.2013.00004

**Published:** 2013-03-20

**Authors:** Idil Arslan-Alaton, Tugba Olmez-Hanci, Bora Genç, Duygu Dursun

**Affiliations:** Department of Environmental Engineering, Faculty of Civil Engineering, Istanbul Technical UniversityIstanbul, Turkey

**Keywords:** advanced oxidation processes (AOPs), persulfate/UV-C process, hydrogen peroxide/UV-C process, nonionic surfactant, octylphenol polyethoxylate, hydroxyl radical, sulfate radical, competitive kinetics

## Abstract

This study explored the potential use of a sulfate radical (SO^·−^_4_)-based photochemical oxidation process to treat the commercial nonionic surfactant octylphenol polyethoxylate (OPPE) Triton™ X-45. For this purpose, the effect of initial S_2_O^2−^_8_ (0–5.0 mM) and OPPE (10–100 mg/L) concentrations on OPPE and its organic carbon content (TOC) removal were investigated at an initial reaction pH of 6.5. Results indicated that very fast OPPE degradation (100%) accompanied with high TOC abatement rates (90%) could be achieved for 10 and 20 mg/L aqueous OPPE at elevated S_2_O^2−^_8_ concentrations (≥2.5 mM). S_2_O^2−^_8_/UV-C treatment was still capable of complete OPPE removal up to an initial concentration of 40 mg/L in the presence of 2.5 mM S_2_O^2−^_8_. On the other hand, TOC removal efficiencies dropped down to only 40% under the same reaction conditions. S_2_O^2−^_8_/UV-C oxidation of OPPE was also compared with the relatively well-known and established H_2_O_2_/UV-C oxidation process. Treatment results showed that the performance of S_2_O^2−^_8_/UV-C was comparable to that of H_2_O_2_/UV-C oxidation for the degradation and mineralization of OPPE. In order to elucidate the relative reactivity and selectivity of SO^·−^_4_ and HO^·^, bimolecular reaction rate coefficients of OPPE with SO^·−^_4_ and HO^·^ were determined by employing competition kinetics with aqueous phenol (47 μM) selected as the reference compound. The pseudo-first-order abatement rate coefficient obtained for OPPE during S_2_O^2−^_8_/UV-C oxidation (0.044 min^−1^) was found to be significantly lower than that calculated for phenol (0.397 min^−1^). In the case of H_2_O_2_/UV-C oxidation however, similar pseudo-first-order abatement rate coefficients were obtained for both OPPE (0.087 min^−1^) and phenol (0.140 min^−1^). From the kinetic study, second-order reaction rate coefficients for OPPE with SO^·−^_4_ and HO^·^ were determined as 9.8 × 10^8^ M^−1^ s^−1^ and 4.1 × 10^9^ M^−1^ s^−1^, respectively. The kinetic study also revealed that the selectivity of SO^·−^_4_ was found to be significantly higher than that of HO^·^.

## Introduction

Different categories and types of surfactants are presently used in cleaning formulations, industrial processes and household activities (Schick, 1987; Ding et al., 1999). Among them, alkylphenol ethoxylates belong to the group of commercially important non-ionic surfactants being widely employed for cleaning and wetting purposes as well as additives in some process chemicals (Ying et al., 2002). Biodegradation is usually rather slow and/or incomplete depending upon the molecular structure, presence of aromatic groups, and the length of the aliphatic chain of alkylphenolic surfactants (Staples et al., 2001).

Alkylphenols, short-chain alkylphenol ethoxylates and short-chain alkylphenol carboxyethoxylates are the most known metabolites of alkylphenolic substances being frequently detected in European, North American, as well as Asian soils sediments, sludge, wastewaters and water bodies (Gianfreda et al., 2003; Coniglio et al., 2008). For example, octylphenol (OP) represents an important metabolite in the biodegradation of octylphenol ethoxylates (OPEs), that are discharged into the aquatic environment via sewage and industrial treatment plant effluents (Staples et al., 2001). Owing to their hydrophobic and non-ionic structure, biodegradation is relatively poor and residues may have serious toxic effects in receiving water bodies. The chronic exposure to OP was reported to have detrimental estrogenic effects on aquatic biota; e.g., fish and invertebrates (Nimrod and Benson, 1996; Ferguson et al., 2000).

Recently, several studies have been devoted to the treatment of industrial surfactants. The major drawback of conventional biological processes is their slow rate and poor removal efficiencies (Chen et al., 2005; Karahan et al., 2010). Among alternative treatment options, so-called advanced oxidation processes (AOPs) have been applied to eliminate surfactants efficiently from water (Arslan-Alaton and Olmez-Hanci, 2012; Arslan-Alaton et al., 2012). AOPs are mainly based on the formation and reaction of hydroxyl radicals (HO^·^). These have been frequently studied to degrade and even mineralize organic emerging pollutants in water, because hydroxyl radical-driven oxidation is extremely rapid and almost unselective with a redox potential of 2.8–2.9 eV (Parsons, 2004). Titanium dioxide-mediated heterogeneous photocatalysis (TiO_2_/UV-A), H_2_O_2_/UV-C, Fenton and Photo-Fenton processes, as well as electrochemical treatment, and ozonation have been intensively studied in the recent past (Esplugas et al., 2002). However, these technologies are quite chemical- and energy-intensive; hence, their implementation could be rather costly (Cater et al., 2000). Consequently, the search for more effective and environmentally friendly and feasible treatment processes has to be progressed.

The persulfate anion (S_2_O^2−^_8_) is a strong oxidizing agent with a redox potential of 2.01 eV (Criquet and Leitner, 2009). Upon thermal, chemical, or photochemical activation, it is possible to generate even stronger oxidizing species (redox potential: 2.4–2.6 eV) that is known as the sulfate radical (SO^·−^_4_) (Dogliotti and Hayon, 1967);

(1) $${\text{S}}_{2}{\text{O}}_{8}^{2-}+\text{h}\nu \to 2{\text{SO}}_{4}^{\cdot -}$$

The *in-situ* generation of SO^·−^_4_ from persulfate-mediated treatment processes has become an increasingly popular remediation process that can be applied to a wide range of organic contaminants found in groundwater and soil (Yang et al., 2008; Rastogi et al., 2009; Gao et al., 2012). Several studies have already demonstrated that SO^·−^_4_ are successfully degrading phenols, chlorophenols, polyaromatic hydrocarbons, pesticides, and dyes (Xu et al., 2012). However, treatment with SO^·−^_4_-driven oxidation processes are relatively rare as compared to HO^·^-based AOPs. Moreover, issues related to reaction pathway, still need to be addressed.

Considering the above indicated facts, the present study focused on the oxidation of the nonionic surfactant octylphenol polyethoxylate, abbreviated as OPPE and commercially known as Triton™-X45, with the persulfate/UV-C oxidation processes. The effect of initial oxidant and pollutant concentration on OPPE and its total organic carbon (TOC) removal rates was explored, followed by comparison with the relatively well-known and established H_2_O_2_/UV-C oxidation process. Finally, the second-order reaction rate coefficient of OPPE with HO^·^ and SO^·−^_4_ were determined by employing competitive kinetics by using phenol as the reference compound. This was done to enable the critical comparison of the relative reactivities and selectivities of these two important oxidizing species.

## Materials and methods

### Materials

**Figure 1:**
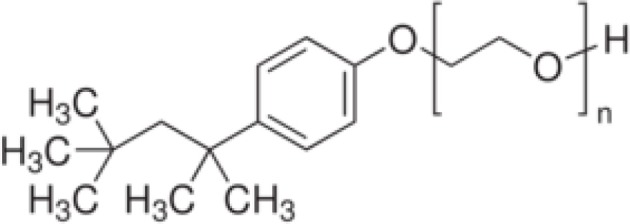
**Molecular structure of Triton™ X-45, *n* ≈ 4.5 (http://www.sigmaaldrich.com)**.

OPPE (Triton™-X45) was purchased Merck 98%; CAS 9002-93-1, Germany) and used without further purification. Triton™-X45 is a mixture of OPEOs with an average ethoxylate chain length of 4.5. The chemical structure of Triton™-X45 is shown in Figure 1. Phenol was purchased from Merck (99%; Germany) and used as received. Potassium persulfate (K_2_S_2_O_8_, ≥99.5%) was purchased from Sigma-Aldrich, Inc. (USA) and H_2_O_2_ (35%, w/w) from Merck (Germany). Aqueous OPPE solutions were prepared in distilled water (Arium 61316RO, Sartorius AG, Germany), whereas for the preparation of the high performance liquid chromatography (HPLC) mobile phase and standard HPLC calibration solutions doubly distilled water (Arium 611UV system, Sartorius AG, Germany) was used.

### Experimental set-up and procedures

S_2_O^2−^_8_/UV-C and H_2_O_2_/UV-C oxidation experiments were conducted at room temperature (25 ± 2°C) in a 1900 mL capacity cylindrical batch photoreactor covered with stainless steel. The UV-C photoreactor setup featuring the UV-C light source (λ_max_ = 253.7 nm) and the procedure of a typical experimental run were previously described elsewhere in more detail (Olmez-Hanci et al., 2011). Samples were taken at regular time intervals for up to 120 min and analyzed for OPPE, TOC and pH. The second-order rate coefficients for the reaction of OPPE with HO^·^ and SO^·−^_4_ were determined through competition kinetics with phenol (47 μM) as the reference compound.

### Analytical measurements

The analysis of OPPE and phenol was performed on a HPLC (Agilent 1100 Series, Agilent Technologies, USA) equipped with a diode array detector (DAD; G1315A, Agilent Series) and a Novapack C18 (3.9 mm × 150 mm, Waters, USA) reversed phase column. The detection wavelength and column temperature were set at 225 nm and 25°C, respectively. The mobile phase consisted of acetonitrile/0.01% phosphoric acid in water (65:35, v/v) at a flow rate of 1.0 mL/min. The quantification limit of OPPE for 50 μL injection volume was calculated as 0.29 mg/L. The mobile phase used for phenol quantification consisted of water/methanol/acetic acid (79.2/19.8/1; v/v/v) at a flow rate of 0.8 mL/min. Diode array detection was performed at 270 nm. The column temperature and injection volume were set as 30°C and 40 μL, respectively. The quantification limit for phenol was determined as 1.5 mg/L for the method described above. The quantification limits of OPPE and phenol were determined by the external standardization method. The correlation coefficients of the established calibration curves for OPPE and phenol were found as 0.999 and 0.998, respectively.

TOC analyses were carried out using a Shimadzu V_CPN_ model carbon analyzer (combustion method) equipped with an autosampler. The detection and quantification limits of the TOC instrument were 50 μg/L and 0.5 mg/L, respectively. The pH was measured using a pH meter (Thermo Orion 720A+) equipped with an Orion model pH electrode (9102BN).

## Results and discussion

### Effect of initial persulfate concentration

**Figure 2:**
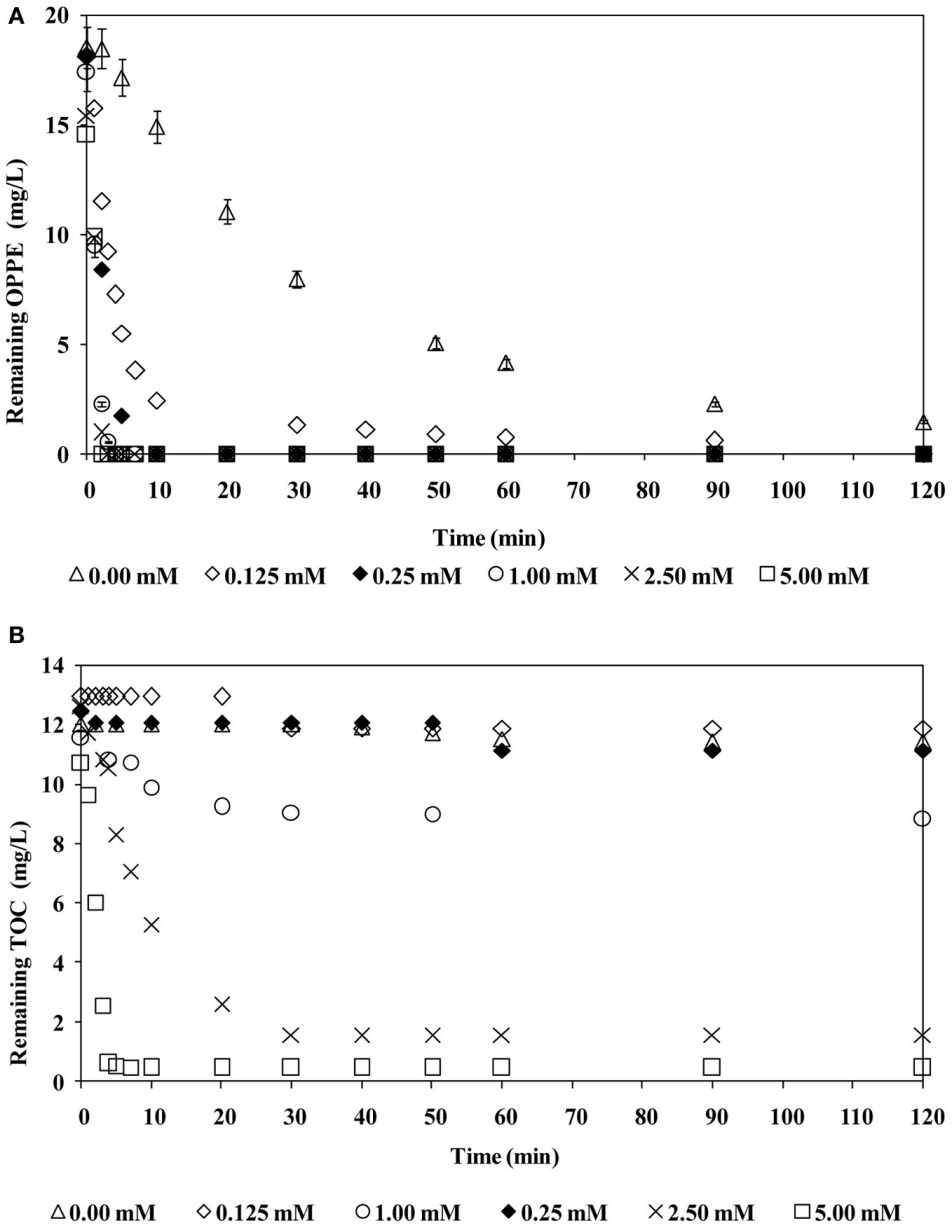
**Effect of initial persulfate concentration on OPPE (A) and TOC (B) abatement rates.** Experimental conditions: Initial pH = 6.5; Initial OPPE = 20 mg/L; Initial TOC = 12 mg/L.

Former studies have already demonstrated that a case-specific optimum oxidant concentration has to be applied to maximize the treatment performance of the H_2_O_2_/UV-C oxidation processes; if H_2_O_2_ is provided insufficiently or excessively, poor removal efficiencies are expected as a consequence of incomplete degradation or competition of the oxidant and the model pollutant for HO^·^, known as the radical scavenging effect (Buxton et al., 1988). The same behavior may be expected for SO^·−^_4_-based AOPs including the persulfate/UV-C treatment process. Figure 2 presents OPPE (a) and TOC (b) removal rates during persulfate/UV-C treatment at varying initial persulfate concentrations (0–5.0 mM) and an initial pH of 6.5. From Figure 2 it is evident that even in the absence of persulfate, direct UV-C photolysis is capable of degrading OPPE; after 120 min treatment with UV-C only, 93% OPPE removal was achieved. Its organic carbon content however, remained practically negligible within this time period (≤5%). From this finding it can be inferred that for the effective treatment of OPPE and its degradation products, persulfate addition was essential. OPPE degradation rates were very fast and OPPE removal was always complete at all studied persulfate concentrations. Elevation of the persulfate concentration in the range of 0.125–1.0 mM resulted in a considerable enhancement in the OPPE removal rate. However, increasing the persulfate concentration ≥1.0 mM had no effect on OPPE degradation rates. At 1.0 mM persulfate concentration, OPPE removal was complete after 3–4 min persulfate/UV-C treatment. Increasing the applied persulfate concentration also had a significant effect on TOC removal efficiencies; overall TOC removals increased from only 8% with 0.125 mM persulfate to 96% when 5.0 mM persulfate was added to the reaction solution. The obtained findings revealed that the presence of a sufficient concentration of persulfate was important for the oxidation of OPPE's degradation products and ultimate oxidation (mineralization). The applied persulfate concentration required careful optimization to ensure effective treatment and minimum chemical costs.

### Effect of initial OPPE concentration

**Figure 3:**
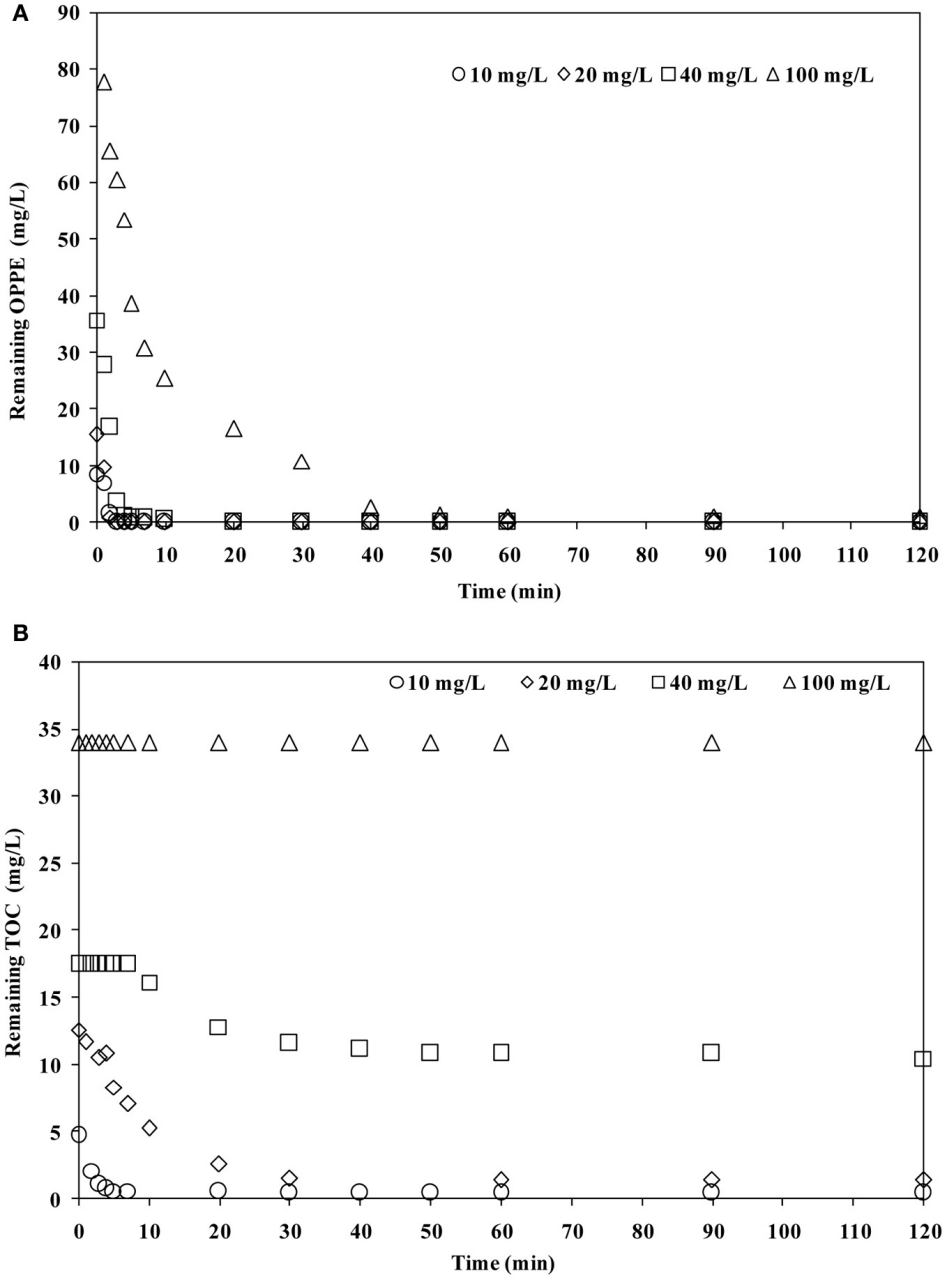
**Effect of initial OPPE concentration on OPPE (A) and TOC (B) abatement rates.** Experimental conditions: Initial pH = 6.5; Initial S_2_O^2−^_8_ = 2.5 mM.

The effect of increasing the OPPE concentration up to its solubility limit at room temperature (from 10 mg/L to ≈100 mg/L) was also investigated at an initial pH of 6.5 and an initial persulfate concentration of 2.5 mM. Results obtained in terms of OPPE (a) and TOC (b) abatement rates are displayed in Figure 3. As is evident in Figure 3, under all circumstances OPPE removal was complete (99–100%) in <40 min under the studied treatment conditions, although removal rates were seriously retarded upon increasing the initial OPPE concentration thereby keeping the oxidant concentration (2.5 mM persulfate) constant. The inhibitory effect was also evident and even more dramatic on the basis of TOC abatements; TOC removal rates were significantly delayed and decreased from 90% for 10 mg/L OPPE down to 40% for 40 mg/L OPPE after 120 min treatment. When the initial OPPE concentration was increased to 100 mg/L, no TOC removal was observed that is thought to be a consequence of the lack of sufficient oxidant to keep up with increasing pollutant (the parent compound OPPE and its degradation products) concentrations. At higher OPPE concentrations, the lack of oxidant may result in secondary reactions, such as accumulation and dimerization of intermediates as well as their competition of with OPPE for SO^·−^_4_ which will affect the OPPE degradation rate dramatically. From these findings it is clear that the removal of higher OPPE concentrations the applied persulfate concentration needs to be elevated in parallel. This is in particular important if mineralization of OPPE is aimed.

### Comparison with H_2_O_2_/UV-C treatment

**Figure 4:**
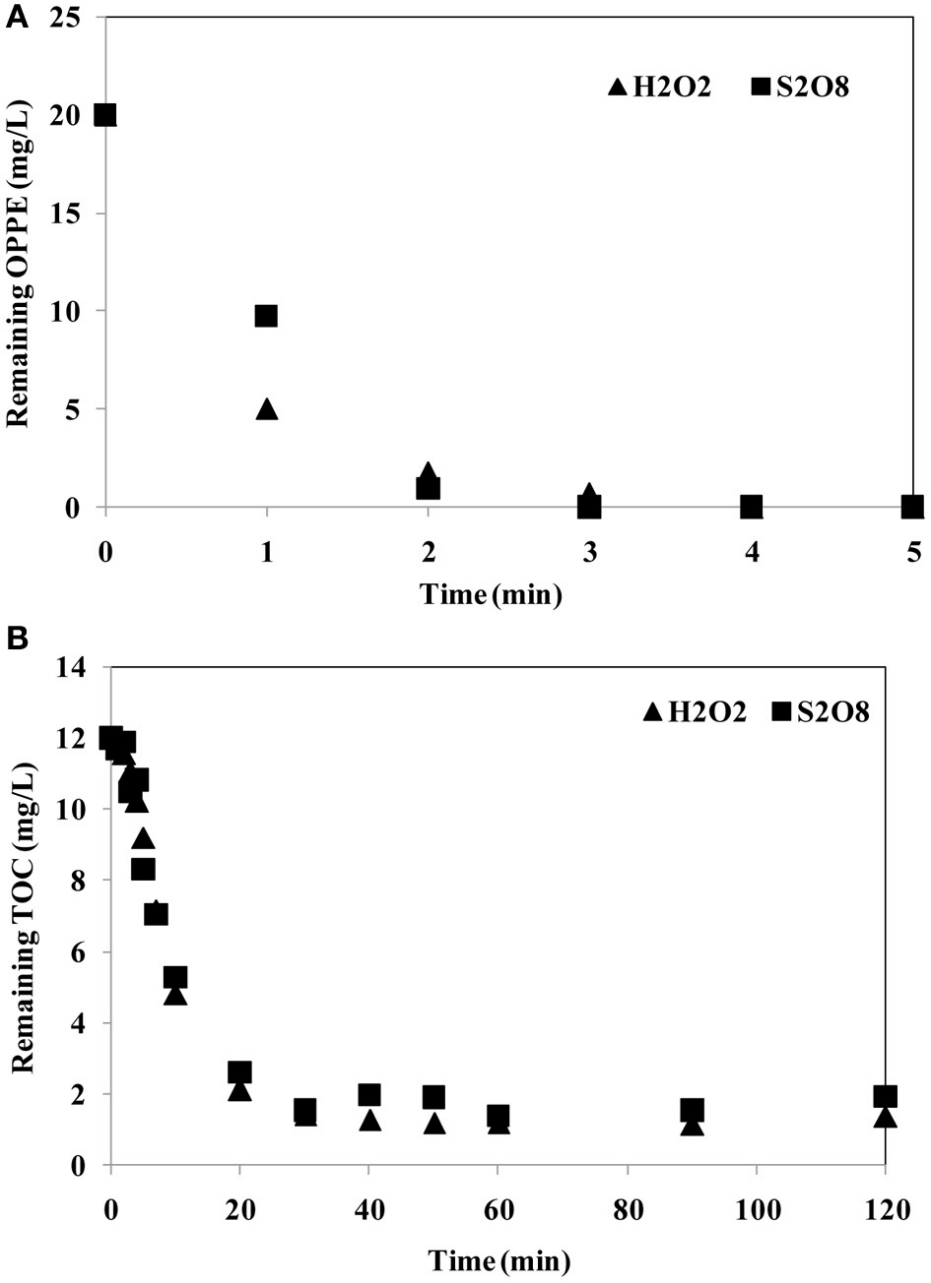
**Comparison of OPPE (A) and TOC (B) abatements during Persulfate/UV-C and Hydrogen peroxide/UV-C treatment.** Experimental conditions: Initial pH = 6.5; Initial oxidant concentration = 2.5 mM; Initial OPPE = 20 mg/L; Initial TOC = 12 mg/L.

S_2_O^2−^_8_/UV-C treatment has intentionally been explored as an alternative to the already well-known and established H_2_O_2_/UV-C oxidation process, that suffers from several limitations as many other AOPs do (Cater et al., 2000). Within the scope of the present study, aqueous OPPE was also subjected to H_2_O_2_/UV-C treatment for comparative purposes. Experimental conditions were selected as 2.5 mM initial oxidant concentration, an initial pH of 6.5 and 20 mg/L aqueous OPPE. Figure 4 comparatively illustrates changes in OPPE (a) and TOC (b) concentrations during S_2_O^2−^_8_/UV-C and H_2_O_2_/UV-C treatment. From Figure 4A it can be deduced that OPPE degradation was complete within 2–3 min for both studied oxidation processes. From the OPPE data obtained after one min treatment it is also evident that H_2_O_2_/UV-C oxidation proceeded at an appreciably faster rate, although OPPE degradation was so rapid that the studied treatment processes overlapped each other in the second minutes of photochemical oxidation. TOC abatements followed a parallel trend; a slow-down was observed after a rapid decrease from 20 mg/L to approximately 5 mg/L being observed after 10 min treatment. During the next 30 min, TOC removal stabilized and remained stagnant thereafter until the end of photochemical treatment (40–120 min). The average TOC values being reached after 120 min were found as 1.2 mg/L and 1.7 mg/L for H_2_O_2_/UV-C and S_2_O^2−^_8_/UV-C oxidation, respectively, corresponding to an ultimate TOC removal efficiency of 89% for H_2_O_2_/UV-C and 84% for S_2_O^2−^_8_/UV-C treatment. The obtained results indicated that S_2_O^2−^_8_/UV-treatment was comparable to the more conventional H_2_O_2_/UV-C oxidation process both in terms of OPPE (parent pollutant) and TOC abatement rates.

### Determination of the reaction rate constant of octylphenol polyethoxylate with hydroxyl and sulfate radicals

In the next and final stage of the study it was aimed at comparing the reactivities of sulfate and hydroxyl radicals with OPPE during persulfate/UV-C and hydrogen peroxide/UV-C treatment, respectively. For this purpose, the second-order reaction rate coefficients for the reaction between OPPE with sulfate and hydroxyl radicals was determined by employing the competitive kinetic approach. A reference pollutant (phenol) whose second-order rate coefficients with sulfate and hydroxyl radicals are known, was added to the reaction solution at equimolar concentrations (47 μM) of the model pollutant (OPPE; 20 mg/L-47 μM) and subjected to (1) persulfate/UV-C and (2) hydrogen peroxide/UV-C treatment. For the kinetic experiments a relatively low oxidant concentration (0.125 mM) was selected to enable sufficient OPPE data collection during the treatment experiments.

For the same experiments the initial reaction pH was reduced to pH3 to ensure that sulfate and hydroxyl radicals were dominant in the reaction solution during (1) persulfate/UV-C and (2) hydrogen peroxide/UV-C treatment, respectively. From the measured phenol (reference pollutant) and OPPE (model pollutant) concentrations, two semi-logarithmic plots (lnC_o_/C versus treatment time curves) were established to determine the first-order abatement rate coefficients for phenol and OPPE during (1) persulfate/UV-C and (2) hydrogen peroxide/UV-C treatment.

**Figure 5:**
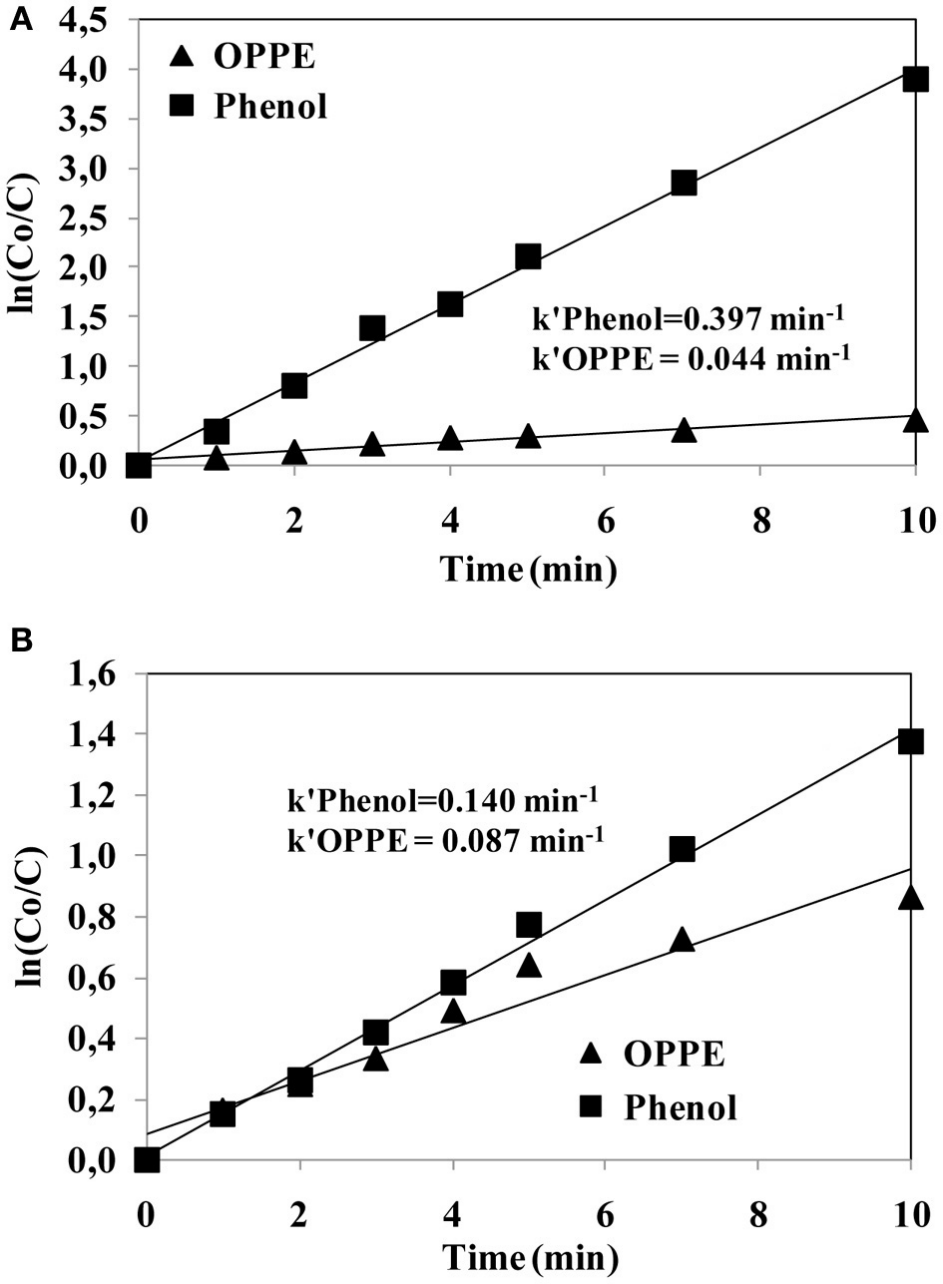
**Comparison of the semi-logarithmic plots of OPPE and phenol abatements with the S_2_O^2−^_8_/UV-C (A) and H_2_O_2_/UV-C (B) treatment processes.** First-order OPPE and phenol abatement rate coefficients are also presented in the figures. Experimental conditions: pH = 3; Initial S_2_O^2−^_8_ and H_2_O_2_ concentrations = 0.125 mM; Initial OPPE (20 mg/L) and phenol molar concentrations = 47 μM.

Figure 5 depicts the semi-logarithmic plots established for OPPE and phenol abatements during persulfate/UV-C (a) and hydrogen peroxide/UV-C (b) treatments. The calculated first-order OPPE (*k*′_OPPE_) and phenol (*k*′_Phenol_) abatement rate coefficients are also shown on the same plot in min^−1^. From the obtained kinetic data for OPPE and phenol it can be inferred that, interestingly, phenol degradation with the persulfate/UV-C process is appreciably (almost three times) faster than its removal with the hydrogen peroxide/UV-C process. On the other hand, the first-order rate coefficient calculated for OPPE removal during persulfate/UV-C oxidation is two times slower than its removal during hydrogen peroxide/UV-C treatment. As such, it can be concluded that the persulfate/UV-C process based on the formation of sulfate radicals is more selective than the hydrogen peroxide/UV-C process that involves hydroxyl radical formation. The used competitive kinetic model is shown below (Benitez et al., 2006);

$$\begin{array}{l} \ln\;{\left[\text{OPPE}\right]}_{\text{o}}/[\text{OPPE}]={\text{k}}_{\text{OPPE},{\text{HO}}^{\cdot }\text{ or }{\text{SO}}_{4}^{\cdot -}}/{\text{k}}_{\text{Phenol},{\text{HO}}^{\cdot }\text{ or }{\text{SO}}_{4}^{\cdot -}}\\ \;\ln{\left[\text{Phenol}\right]}_{\text{o}}/[\text{Phenol}] \end{array}$$

The calculated first-order rate coefficients were inserted to the re-arranged competitive kinetic equations derived for the reaction of OPPE with HO^·^ and SO^·−^_4_ as given below;

(2) $${\text{k}}_{\text{OPPE},\;{\text{HO}}^{\cdot }}=\left({{\text{k}}^{\prime }}_{\text{OPPE}}/{{\text{k}}^{\prime }}_{\text{Phenol}}\right)\times {\text{k}}_{\text{Phenol},\;{\text{HO}}^{\cdot }}$$

and;

(3) $${\text{k}}_{\text{OPPE},\;{\text{SO}}_{4}^{\cdot -}}=\left({{\text{k}}^{\prime }}_{\text{OPPE}}/{{\text{k}}^{\prime }}_{\text{Phenol}}\right)\times {\text{k}}_{\text{Phenol},\;{\text{SO}}_{4}^{\cdot -}}$$

with;

k_Phenol, SO^·^_4__ = second-order reaction rate coefficient of phenol with the sulfate radical (8.8 × 10^9^ M^−1^ s^−1^; Lindsey and Tarr, 2000; Liang and Su, 2009)

and;

k_Phenol, HO^·^_ = second-order reaction rate coefficient of phenol with the hydroxyl radical (6.6 × 10^9^ M^−1^ s^−1^; Lindsey and Tarr, 2000; Liang and Su, 2009)

From Equations (1) and (2) given above the second-order reaction rate coefficients for OPPE with sulfate and hydroxyl radicals were determined as 9.8 × 10^8^ M^−1^ s^−1^ and 4.1 × 10^9^ M^−1^ s^−1^, respectively. Comparing these rate coefficients with those already known for phenol once again implied that the reactivity and selectivity of sulfate radicals differs significantly from that of hydroxyl radicals for the same and different pollutants; it appears that the sulfate radical has a higher selectivity for some pollutants (e.g., prefers phenol over OPPE), whereas the hydroxyl radical exhibits relatively less selectivity toward different organic pollutants (reacts almost equally fats with phenol and OPPE). This issue has to be considered seriously when adopting and selecting an appropriate oxidative treatment system for the removal of toxic and/or refractory organic pollutants.

## Concluding remarks

In the present study, the oxidation of the commercial non-ionic surfactant octylphenol polyethoxylate (OPPE) with the S_2_O^2−^_8_/UV-C was investigated and compared to its treatability with the more conventional H_2_O_2_/UV-C process. The following conclusions could be drawn from the present work;

- Increasing the initial S_2_O^2−^_8_ concentration improves OPPE and TOC removals appreciably.
- Increasing the initial OPPE concentration negatively affected in particular TOC removals due to insufficient oxidant (SO^·−^_4_) supply.
- The treatment performance of S_2_O^2−^_8_/UV-C oxidation was found to be very close to that of the H_2_O_2_/UV-C process proceeding only slightly faster in terms of OPPE removal, speaking for some differences in reaction kinetics and selectivity.
- The second-order reaction rate coefficients between OPPE and SO^·−^_4_ (9.8 × 10^8^ M^−1^ s^−1^) as well as HO^·^ (4.1 × 10^9^ M^−1^ s^−1^) were determined by employing competitive kinetics using phenol as the reference compound.
- SO^·−^_4_ was found to be more selective than HO^·^ according to competitive phenol and OPPE abatements.

### Conflict of interest statement

The authors declare that the research was conducted in the absence of any commercial or financial relationships that could be construed as a potential conflict of interest.
